# Black Soldier Fly (*Hermetia illucens*) Larvae and Prepupae Defatted Meals in Diets for Zebrafish (*Danio rerio*)

**DOI:** 10.3390/ani11030720

**Published:** 2021-03-06

**Authors:** Carlos F. C. Lanes, Fabio A. Pedron, Giovani T. Bergamin, Andressa L. Bitencourt, Brenda E. R. Dorneles, Jessica C. V. Villanova, Kimberly C. Dias, Kristian Riolo, Sabrina Oliva, Domenico Savastano, Alessia Giannetto

**Affiliations:** 1 Curso Superior de Tecnologia em Aquicultura, Campus Uruguaiana, Federal University of Pampa, Uruguaiana, Rio Grande do Sul 118, Brazil; carloslanes@unipampa.edu.br (C.F.C.L.); fabiopedron@unipampa.edu.br (F.A.P.); giovanibergamin@unipampa.edu.br (G.T.B.); andressabitencourt.aluno@unipampa.edu.br (A.L.B.); brendadorneles.aluno@unipampa.edu.br (B.E.R.D.); jessicavillanova.aluno@unipampa.edu.br (J.C.V.V.); kimberlydias.aluno@unipampa.edu.br (K.C.D.); 2 Department of Chemical, Biological, Pharmaceutical and Environmental Sciences, University of Messina, Viale F. Stagno d’Alcontres n. 31, 98166 Messina, Italy; kristian.riolo@unime.it (K.R.); soliva@unime.it (S.O.); 3 Progetto Hermetia, 89013 Gioia Tauro, Italy; domenicosavastano@hotmail.it

**Keywords:** aquaculture, insect meal, *Hermetia illucens*, gene expression, chitin, fishmeal, fish wellness

## Abstract

**Simple Summary:**

Aquaculture accounts for 52% of the world’s production of fish for human consumption and its growth relies on finding new alternative protein sources to fishmeal to use in aquafeeds. To make fish feed more sustainable, insects have emerged as valuable alternatives due to their high nutritional value. In this context, black soldier fly (BSF) meals have been used to replace, partially or totally, fishmeal in aquafeeds. However, due to relevant nutritional peculiarities of BSF developmental stages, it is crucial to understand how they can affect fish growth performance and health status. In this study, we evaluated the effects of total dietary replacement of fishmeal with defatted BSF larvae and prepupae meals on the growth performance of zebrafish from larva, through adult stages, in a 60-day feeding trial. Moreover, the effects of the two BSF meals on the expression of genes involved in growth, stress- and immune- responses, as well as hydrolysis of chitin were investigated. Overall, our results showed the beneficial role of defatted BSF meals in zebrafish diet, especially regarding prepupae meal, suggesting that black soldier fly meals could totally replace fishmeal without negatively affecting growth and wellness of zebrafish.

**Abstract:**

The black soldier fly (BSF) *Hermetia illucens* is receiving increasing attention as a sustainable fishmeal alternative protein source for aquaculture. To date, no studies have explored the effects of fishmeal replacement with BSF V instar larvae or prepupae meals due to their peculiar nutritional properties on fish performances. This study investigated the effects of 100% replacement of fishmeal (control diet) with defatted BSF meals (V instar larvae and prepupae meals, treatments) on growth performance and welfare of zebrafish (*Danio rerio*), from larvae to adults, in a 60-day feeding trial. Following the inclusion of BSF meals, the expression of key genes involved in growth (*igf1*, *igf2*, *mstnb*, *myod1*, *myog*, *myf5*), hydrolysis of chitin (*chia.2*, *chia.3*, *chia.5*), immune- (*il1b*, *il6*, *tnfα*), and stress- (*hsp70* and *nr3c1*) responses, as assessed by qPCR, was modulated in all of the molecular pathways, except for the stress response. Overall, our findings showed that both BSF meals can totally replace fishmeal without adverse impacts on adult zebrafish growth parameters (final total and standard length, final body weight, weight gain, daily growth rate, specific growth rate) and welfare, with BSF prepupae meal inducing the most beneficial effects, thus suggesting their potential application to meet fish requirements in aquaculture.

## 1. Introduction

Over the last three decades, the aquaculture industry has been the world’s fastest growing food-producing sector. In 2018, aquaculture production reached 82.10 million tons, representing 52% of the world’s fish used for human consumption [1]. The future growth of aquaculture relies on the finding of sustainable alternative protein sources to use in aquafeeds. Fishmeal has been largely used as one of the primary protein source for farmed fish feeds due to its highly digestible protein, properly balanced amino acids profile, good palatability, as well as, deficient in the anti-nutritional factors [2,3]. However, both environmental and economic (decreased availability and increased cost) concerns increase the urgent need to replace fishmeal for sustaining the further development of aquaculture. Currently, plant-based proteins (especially soybean meal) and processed animal proteins (PAPs) from non-ruminant animals (poultry and pigs) are used as ingredients in formulated fish feeds, to meet the fish’s nutritional requirements for their good digestibility and palatability, lower carbon footprint and reduced levels of anti-nutritional factors than vegetable products, which improves fish health and welfare. Among the fishmeal alternative protein sources, PAP from insect species has been gaining increasing attention for the valuable nutritional properties of insect larvae meals [4].

Recently, the European Commission (Regulation 2017/893/EC) approved seven species of insects to be used in aquafeeds: housefly (*Musca domestica*), black soldier fly (*Hermetia illucens*), beetles (*Tenebrio molitor* and *Alphitobius diaperinus*), and crickets (*Ancheta domesticus*, *Gryllodes sigillatus,* and *Gryllus assimilis*). In 2018, the United States released the use of black soldier fly as food for salmonids [5].

The production of insects as protein source for aquafeeds shows a series of interesting and eco-friendly characteristics. For example, insects can efficiently bioconvert low-grade organic substrates into high-quality protein. They do not require arable land, which allows the production of protein in infertile or degraded soils. They do not compete with human food sources or human food production. They have short life cycles and can grow on a wide range of substrates with high productivity. They have low demand for water and energy when compared to other livestock; and insects have a low risk of transmitting zoonotic infections [4,6,7,8]. 

Among the promising insect species investigated as alternative nutrient sources for aquafeeds, so far, the black soldier fly (BSF) has been identified as a suitable candidate. Previous studies have assessed the effects of fishmeal replacement (partial or total) with BSF meals in aquafeeds [9,10,11,12,13]. Although BSF inclusion in fish diets has shown favorable results, no scientific reports established the most suitable BSF larval stage to be used as feed until now. Recently, relevant differences in the proximate composition of BSF V instar larvae and prepupae, in terms of both fatty acid and amino acid profiles, as well as minerals and vitamins, have been demonstrated [14,15,16]. In addition, Huyben et al. [17] evaluated the differences in gut microbiota of rainbow trout (*Oncorhynchus mykiss*) fed with BSF larvae or prepupae, demonstrating that the gut bacteria composition in this species is also influenced by the developmental stage of the insect used as meal.

Zebrafish (*Danio rerio*) has been widely used as an experimental model in several areas, including neurosciences, toxicology, genetics, embryology, metabolism, and oncology. In aquaculture field, zebrafish has been used in nutritional studies as an ideal alternative to commercial fish species for preliminary assessments of different novel ingredients late suggested as suitable for aquafeeds [18]. Moreover, zebrafish has been considered as an ideal platform for nutritional genomic studies due to the availability of a wide range of molecular tools [19].

The present study aimed to investigate the effects of total dietary replacement of fishmeal with defatted BSF larvae and prepupae meals on zebrafish growth performance and welfare. The expression of key genes involved in fish growth (*igf-1*, *igf-2*, *mstnb*, *myod1*, *myog*, *myf5*), enzymatic hydrolysis of chitin (*chia.2*, *chia.3*, *chia.5*), immune (*il1b*, *il6*, *tnfα*), and stress responses (*hsp70 and nr3c1*) was evaluated to understand the physiological and molecular responses to the inclusion of BSF meals in the zebrafish diet. Such information might be helpful to select the most suitable BSF developmental stage for future inclusion of BSF in the aquafeeds.

## 2. Materials and Methods

### 2.1. Insect Source

Specimens of *Hermetia illucens* were bred and collected from an established colony (www.progettohermetia.it) (Gioia Tauro, Italy). After hatching, BSF larvae were put on a mix of flour (35%) and water (65%) for 5 days and then reared on organic waste composed of a mix of vegetable and fruit wastes (40% pears, 45% banana, 5% tomatoes, 10% various leafy green vegetables, % *w*/*w* on fresh weight basis) as described by Giannetto et al. [15]. The substrate was ground with a 3 mm die meat mincer (Fama Industrie, Rimini, Italy) before being used to feed the larvae. The V instar larvae and prepupae were recognized following established criteria by Kim et al. [20] and Tomberlin et al. [21]. The V instar larvae and prepupae samples were collected and set at 4 °C to allow quiescence to be reached. Samples were dried at 55 °C in a ventilated oven (TE-394/2-MP, Tecnal, Piracicaba/SP, Brazil) until a constant weight and ground. The proximate composition of V instar and prepupae was previously reported in Giannetto et al. [15].

### 2.2. Experimental Diets

Dried V instar larvae and prepupae samples were crushed and defatted with hexane (4 L of reagent to 1 kg of material). After 30 min, hexane was removed and the material was dried during 24 h at room temperature (≅20 °C). Afterwards, the samples and all ingredients were milled in a small size hammer mill and sieved using a 150 μm mesh sieve.

Experimental diets are shown in Table 1. The diets were formulated to be isonitrogenous and isolipidic, according to zebrafish requirements [22]. The control diet (Ctr) contained fishmeal (processing residue) as a main protein source. In the other two experimental diets, fishmeal was replaced with 100% of defatted V instar larvae meal (V instar) or with 100% defatted prepupae meal (prepupae). To prepare the experimental feeds, the milled dry ingredients (<150 μm) and oils were thoroughly mixed manually. Water was added to the mixtures to attain an appropriate consistency for pelleting. The feeds were pressed through a meat grinder (PBM10I, Beccaro, Rio Claro/SP, Brazil) that had a 3.5 mm die and dried at 50 °C for 24 h in a ventilated oven (TE-394/2-MP, Tecnal, Piracicaba/SP, Brazil) and subsequently stored at 4 °C.

The proximate nutrient composition in the final feeds was analyzed to inspect whether the nutrient composition was as expected, based on calculations (Table 1). Dry matter, ash, and protein content were determined according to Association of Official Analytical Chemists AOAC [23]. Total fat content was determined gravimetrically following the method of Bligh and Dyer [24]. Acid detergent fiber (ADF) was determined by the method of Van Soest et al. [25]. The chitin content was estimated by calculating the acid detergent fibers (ADFs) and subtracting to this value the amino acids (by weight) contained in the ADF residue (%), as described by Finke [26]. All of the chemical analyses were carried out in triplicate.

### 2.3. Fish Feeding Trial and Growth Analysis

Eggs of zebrafish were obtained from a broodstock maintained at the Laboratory of Ornamental Fish from Federal University of Pampa—Campus Uruguaiana under the conditions described by Westerfield [27]. At 30 days post-hatching (dph), larvae (5.0 ± 0.5 mg; 9.2 ± 0.3 mm, mean ± standard error) were randomly distributed in twelve 4 L tanks (25 larvae per tank), with four replicate tanks for each treatment (Ctr, V instar, and prepupae) in a recirculating water system. Water temperature and dissolved oxygen were monitored daily while other water quality parameters were monitored weekly. Temperature was kept at 27 ± 0.3 °C, pH = 8.1 ± 0.1, dissolved oxygen = 6.4 ± 0.12 mg/L and 12L:12D photoperiod.

Fish were fed with experimental diets three times a day (8:00 a.m., 12:00 p.m., and 5:00 p.m.) for 60 days ad libitum. The tanks were siphoned once daily at 11:00 a.m. and the water was replenished. From days 0 to 15, fish were fed with food particle size <150 μm. From days 16 to 35, fish were fed with food particle size 150–250 μm. Thereafter, fish were fed with food particle size 300–400 μm. The total biomass in each tank was evaluated to each 15 days to evaluate the growth. For this, all fish were anesthetized with eugenol (60 mg/L) [28]. During the experiment, the dead fish were removed and recorded for calculating the survival rate. The survival rate was calculated considering the remaining individuals in each tank, discounting the two fish that were sampled in the middle of experiment from each tank. At the end of the experiment, the animals were individually anesthetized, weighted, and photographed with a digital camera. The total length, standard length and height were taken from each image through the open-source software IMAGE J (US National Institute of Health, available at http://rsb.info.nih.gov/ij/, accessed on 29 August 2019). The growth parameters were calculated using the following equations:

Weight gain (WG) = final fish weight (W_f_) − initial fish weight (W_i_);
Daily growth rate (DGR) = (W_f_ − W_i_)/T, where T represents the time of the study in days;
Specific growth rate (SGR) = ((ln W_f_ − ln W_i_/T)) × 100;
Condition factor index (K) = (W × L^−3^) × 10^3^, where W is mass in milligrams and L is standard length in millimeters.

### 2.4. RNA Extraction and cDNA Synthesis

All samples were collected at each sampling time, prior to morning feeding. For quantitative PCR assays, eight pooled samples of ten larvae were taken at the beginning of the experiment, and two juveniles (60 dph) from each tank (eight specimens per treatment) were taken after 30 days of experiment. At the end of experiment (60 days of experiment), three adult animals (90 dph) from each tank (12 specimens per treatment) were randomly sampled and sacrificed for collecting the liver (to assess the expression of the genes related to immune and stress responses), muscle (to assess the expression of the genes related to muscle growth), and gut (to assess the expression of genes related to chitin hydrolysis) for subsequent extraction of total RNA. All samples were stored in *RNAlater* (Sigma-Aldrich Milano, Italy) according to manufacturer’s instructions. Total RNA was extracted from samples using QIAzol lysis reagent (Qiagen, Milano, Italy), according to manufacturer’s instructions. RNase-free DNase (Qiagen, Milano, Italy) was used to eliminate contaminating genomic DNA. RNA quantity, purity, and integrity were verified by both electrophoresis on 1.0% agarose gel and UV absorbance measurements at 260 and 280 nm (NanoDrop 2000, Thermo Scientific, Wilmingtom, MA). One microgram total RNA was reverse transcribed into cDNA by QuantiTect reverse transcription kit (Qiagen) according to the manufacturer’s protocol.

### 2.5. Quantitative Gene Expression (qPCR)

The cDNA samples were used to evaluate the mRNA levels of selected genes associated with different pathways by quantitative real time PCR. Transcripts of the genes related to muscular growth, namely *insulin-like growth factor 1* (*igf-1*) and *2* (*igf-2*), *myostatin b* (*mstnb*), *myogenic differentiation 1* (*myod1*), *myogenin* (*myog*) and *myogenic factor 5* (*myf5*), to immune and stress response—*interleukin 1 beta* (*il1b*), *interleukin 6* (*il6*), *tumor necrosis factor α* (*tnfα*), *heat-shock protein-70* (*hsp70*), *glucocorticoid receptor* (*nr3c1*)—as well as genes involved in the enzymatic hydrolysis of chitin—*chitinase 2*, *3* and *5* (*chia.2*, *chia.3*, *chia.5*) were quantified using specific qPCR primer sets (Table 2).

PCR reactions were performed with SYBER Green chemistry (Qiagen) in the Rotor-Gene Q 2plex Hrm thermocycler (Qiagen) and twenty-fold diluted cDNA samples (six biological replicates for each experimental diet and sampling time) were run in duplicate together with no template and minus reverse transcriptase controls. The PCR efficiency was determined performing, for each gene, a five-point standard curve of a 5-fold dilution series (1:1 to 1:32) from pooled RNA as described by Giannetto et al. [29]. The normalization factor was calculated by the geNorm software (http://medgen.ugent.be/~jvdesomp/genorm/, accessed on 16 October 2020) from the two most stable reference genes among *acid ribosomal phosphoprotein* (*arp*), *ribosomal protein L13* (*rpl13*), and *elongation factor 1α* (*ef1-α*) in order to correct the raw target gene data, as described by Nagasawa et al. [30]. The specificity of each reaction was evaluated by dissociation curve analysis that showed a single pick in each run.

### 2.6. Statistical Analysis

Growth parameters data were subjected to one-way analysis of variance (ANOVA). Post-hoc comparisons were made by Tukey’s test. Data were expressed as mean ± S.E. The differences in transcript levels of tested genes among the experimental groups of fish fed with the three diets were assessed by analysis of variance followed by Student–Newman–Keuls post-hoc tests. Normality and variance homogeneity were previously checked and a significance level of 5% was adopted in every case. Data were expressed as mean ± S.D. Statistical analyses were performed using the SAS software [31].

## 3. Results

### 3.1. Survival and Growth Performance

At the end of the experiment, no significant differences were observed in the survival rates among the dietary treatments (*p* > 0.05; Table 3). All feeds were utilized in the trial initially floated and were readily consumed by zebrafish.

During the first 30 days of experiment, there were no weight differences among treatments (*p* > 0.05; Figure S1). However, at the end of the experiment, significant differences were observed in all growth parameters among the treatments. Zebrafish fed with prepupae treatment had significantly higher total length, standard length, height, final body weight, weight gain, daily growth rate, and specific growth rate compared to control treatment (*p* < 0.05; Table 3), but no significant difference when compared to V instar treatment (*p* > 0.05; Table 3) was found. For all of these parameters, no significant differences were observed between V instar and control (*p* > 0.05; Table 3). Regarding the condition factor index, fish from control group showed a higher value than V instar group, but no differences were observed between control and prepupae treatment (Table 3).

### 3.2. Quantitative Gene Expression

#### 3.2.1. Muscle Growth

**Figure 1:**
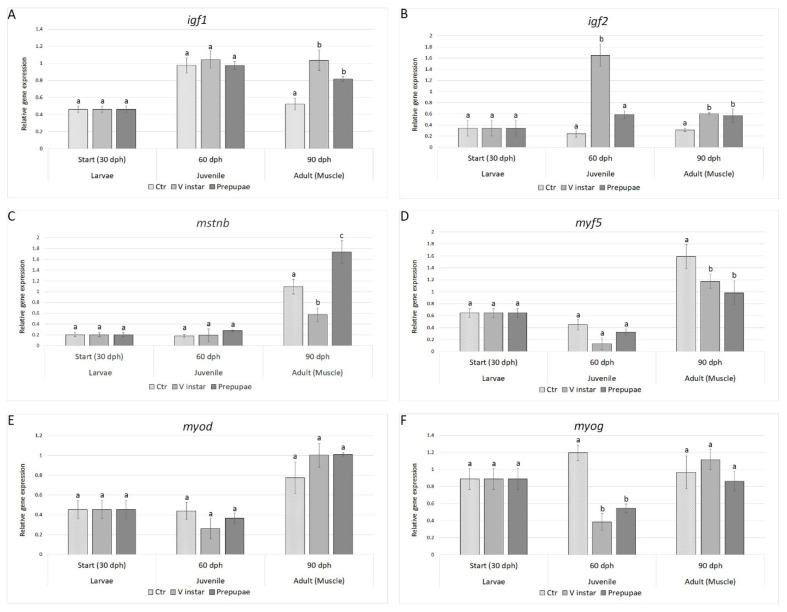
Expression profiles of genes involved in growth: (**A**) *igf1*, (**B**) *igf2*, (**C**) *mstnb*, (**D**) *myf5*, (**E**) *myod*, and (**F**) *myog*. Relative mRNA levels were evaluated in zebrafish larvae (30 dph), juveniles (60 dph), and adults (90 dph) fed with V instar and prepupae diets compared to controls fed with a diet containing fishmeal as the main protein source. Data are expressed as mean ± S.D. (*n* = 6). Different letters indicate significant differences in transcript levels among experimental groups within the same sampling time (*p* < 0.05).

Quantitative gene expression analysis showed that the transcript levels of the genes involved in fish growth were peculiarly modulated by the two BSF diets (Figure 1). The major effects of BSF inclusion on the gene expression were evident in adult zebrafish (90 dph) and to a lesser extent in juveniles (60 dph).

Although the *igf1* gene expression did not show significant differences among the three experimental groups at 60 dph (juvenile), the different BSF diets significantly influenced the *ifg1* expression at 90 dph (adult) with both V instar and prepupae groups showing significantly higher mRNA levels (2- and 1.6-fold, respectively) with respect to control group (*p* < 0.001; Figure 1A).

V instar meal fed zebrafish showed significantly higher *igf2* gene expression at 60 dph with respect to control (5.3-fold; *p* < 0.05). At 90 dph, both V instar and prepupae groups showed significantly higher *igf2* gene expression (about 2-fold for both treatment groups) with respect to control (*p* < 0.05; Figure 1B).

The *mstnb* transcript levels did not show significant differences in both the experimental groups with respect to control at 60 dph (Figure 1C). On the contrary, at the adult stage, the *mstnb* gene expression levels were significantly lower (1.8-fold) in the V instar group (*p* < 0.05) and significantly higher (1.5-fold) in prepupae group (*p* < 0.05), with respect to control. The opposite trend of *mstnb* gene expression in adults indicated that the different BSF diets could also modulate the levels of this growth factor (Figure 1C).

The *myf5* gene expression pattern did not show significant differences in both treatment groups with respect to control at 60 dph, while both V instar and prepupae groups showed a significant decrease in *myf5* gene expression as compared to control group at 90 dph (*p* < 0.05), with levels 1.3-fold (V instar) and 1.6-fold (prepupae) lower in BSF-fed zebrafish, with respect to specimens fed with control diet (Figure 1D).

The *myod* gene expression did not show significant differences among the three experimental groups at all the sampling times as the transcript levels in both V instar and prepupae groups were comparable to control in zebrafish juvenile and adults (Figure 1E).

Finally, the *myog* gene expression did not show differences in both V instar and prepupae groups, with respect to control at 90 dph (Figure 1F). However, *myog* transcript levels were significantly lower in both experimental groups, with respect to control at 60 dph, with a significant downregulation of *myog* expression in V instar (3-fold) and prepupae groups (2.2-fold), respectively (*p* < 0.05).

#### 3.2.2. Enzymatic Hydrolysis of Chitin

**Figure 2:**
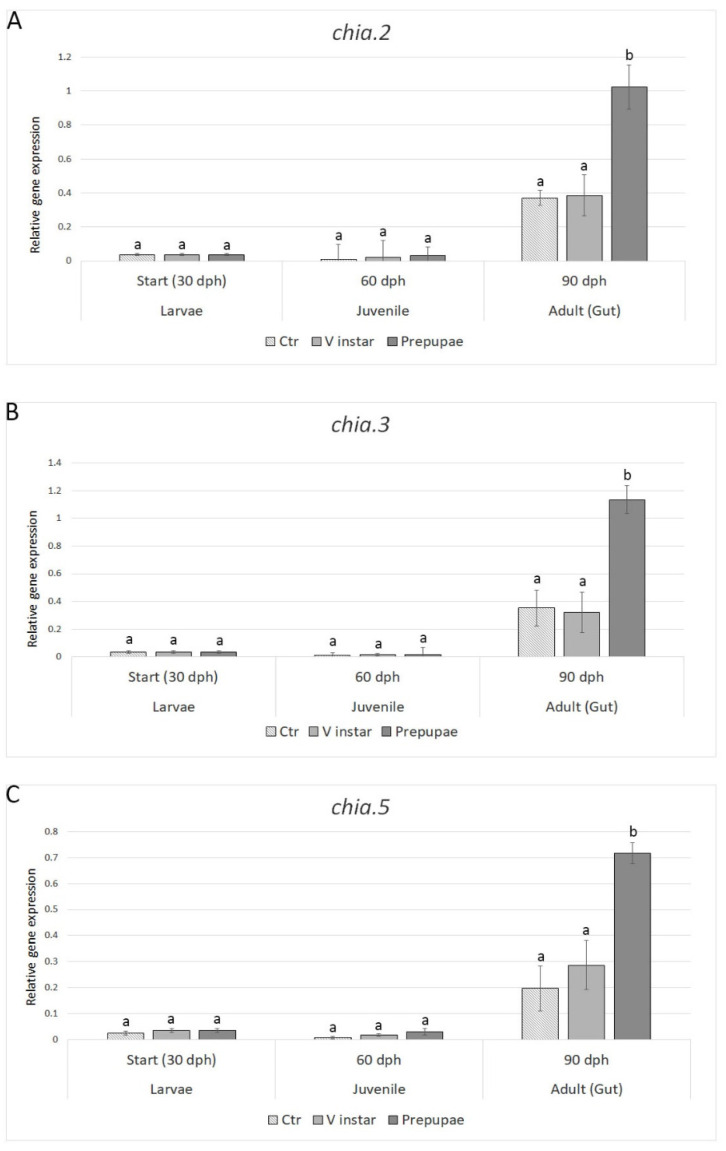
Expression profiles of genes associated with chitin hydrolysis: (**A**) *chia.2*, (**B**) *chia.3*, and (**C**) *chia.5*. Relative mRNA levels were evaluated in zebrafish larvae (30 dph), juveniles (60 dph), and adults (90 dph) fed with V instar and prepupae diets compared to controls fed with a diet containing fishmeal as the main protein source. Data are expressed as mean ± S.D. (*n* = 6). Different letters indicate significant differences in transcript levels among experimental groups within the same sampling time (*p* < 0.05).

The expression of the genes involved in enzymatic hydrolysis of chitin (*chia.2*, *chia.3*, and *chia.5*) was modulated by the different BSF diets exclusively in adult zebrafish (Figure 2). The transcript levels of all the investigated chitinases were significantly upregulated in fish fed BSF-prepupae (*p* < 0.001) with values 2.5- (*chia.2*), 3.1- (*chia.3*), and 3.5-fold (*chia.5*) higher, with respect to control. Noteworthy, V instar BSF diet did not induce significant differences in chitinase genes expression in both juvenile and adult zebrafish.

#### 3.2.3. Immune Response

**Figure 3:**
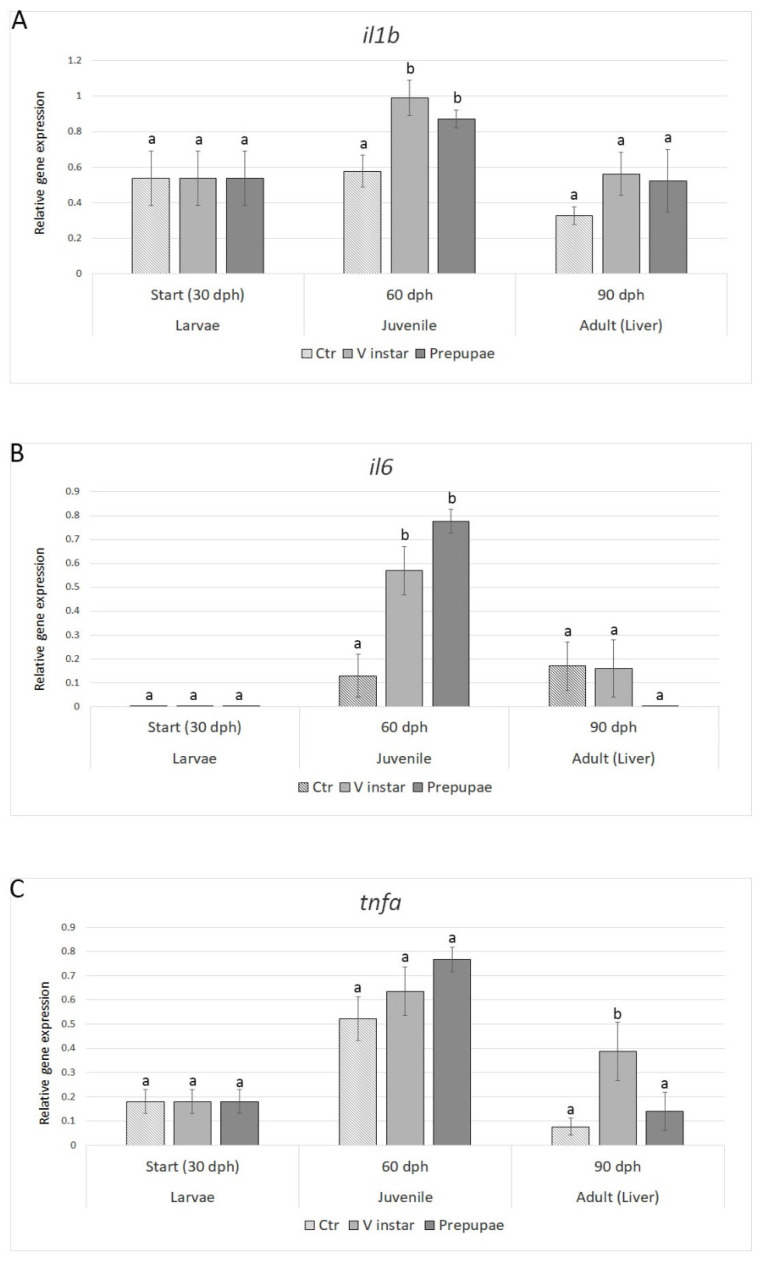
Expression profiles of genes involved in immune response (**A**) *il1b*, (**B**) *il6*, and (**C**) *tnfα*. Relative mRNA levels were evaluated in zebrafish larvae (30 dph), juveniles (60 dph), and adults (90 dph) fed with V instar and prepupae diets compared to controls fed with a diet containing fishmeal as the main protein source. Data are expressed as mean ± S.D. (*n* = 6). Different letters indicate significant differences in transcript levels among experimental groups within the same sampling time (*p* < 0.05).

The analysis of the expression levels of genes involved in immune response (*il1b*, *il6,* and *tnfa*) showed that *il1b* and *il6* were upregulated in both treatment groups (with respect to control) in juveniles, while their transcript levels did not show any significant difference in adults among the three experimental groups (Figure 3A,B). The *tnfa* gene expression was affected by the different BSF diets exclusively in adult zebrafish fed BSF-V instar with values 4-fold higher with respect to control (Figure 3C). Prepupae diet did not affect *tnfa* gene expression in either juveniles or adults.

#### 3.2.4. Stress Response

**Figure 4:**
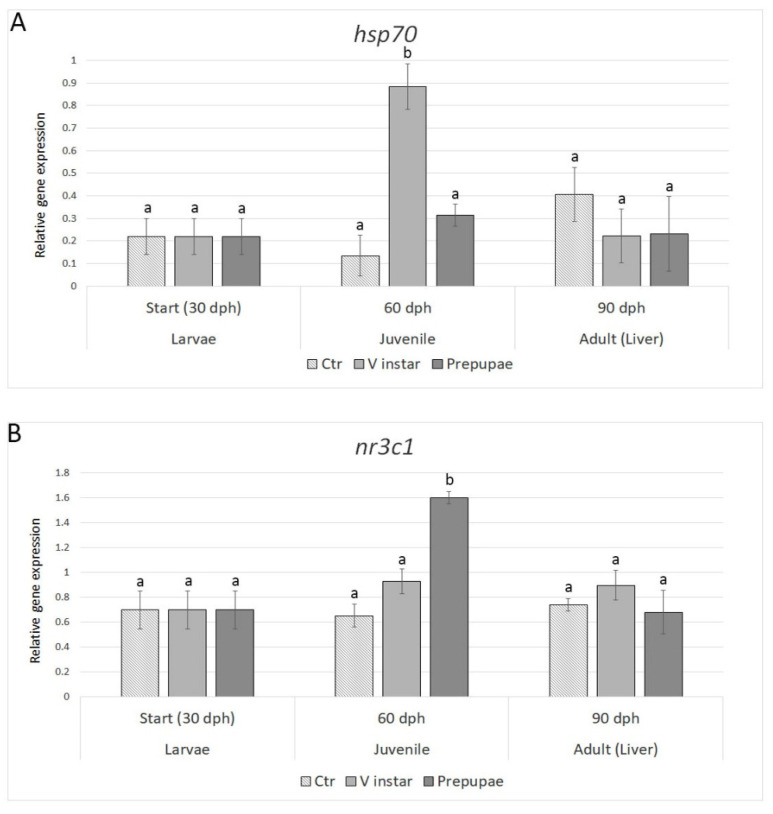
Expression profiles of stress-related genes (**A**) *hsp70* and (**B**) *nr3c1*. Relative mRNA levels were evaluated in zebrafish larvae (30 dph), juveniles (60 dph), and adults (90 dph) fed with V instar and prepupae diets compared to controls fed with a diet containing fishmeal as the main protein source. Data are expressed as mean ± S.D. (*n* = 6). Different letters indicate significant differences in transcript levels among experimental groups within the same sampling time (*p* < 0.05).

Transcript levels of the selected stress markers were modulated by the different BSF diets exclusively in juvenile zebrafish (Figure 4). On the contrary, both *hsp70* and *nr3c1* gene expression levels did not show any significant differences in V instar and prepupae groups, with respect to control in adult fish (90 dph).

## 4. Discussion

Among several insect species tested in aquafeeds, BSF is one of the most promising because it shows an essential amino acid profile similar to fishmeal [4]. This study demonstrated that the total substitution of fishmeal with two different BSF meals (obtained from two BSF developmental stages, i.e., V instar larvae and prepupae) in zebrafish diet do not adversely affect growth performances and wellness. The inclusion of defatted V instar larvae and defatted prepupae meals induced a positive effect on growth parameters. Although recent literature has reported contrasting data on the effect of insect inclusion in fish diets [32], the long-term trial here evaluated showed the beneficial role of defatted BSF meals in zebrafish diet.

Previous studies showed marked differences on fatty acid and amino acid profiles between the larval and prepupae stages in BSF [14,15,16]. Recent studies on zebrafish have demonstrated that diets containing higher levels than 50% of inclusion of full-fat prepupae meal negatively affect the growth and induce hepatic steatosis, microbiota modification, higher lipid content, fatty acid modification, and higher expression of immune response markers [13,33]. According to authors, all of these negative effects could be related to fatty acid imbalance coming from BSF meal. It is known that the quantity of proteins and lipids can vary in BSF according to the rearing conditions, mainly the substrate used to feed the larvae. While the amino acid profile is less affected, the fatty acid composition depends greatly on the feed source [34,35]. The different findings could rely on the defatted BSF meal used in the present study, thus suggesting that this formulated diet can mitigate the imbalance HUFA/SFA (Highly Unsaturated Fatty Acid/Saturated Fatty Acid) characterizing insect meals and the subsequent negative effects previously reported [13,33]. Belghit et al. [10], also working with defatted insect meal, found that fish meal replacement with BSF up to 100% did not determine a decreased growth of Atlantic salmon (*Salmo salar*), suggesting BSF as a good alternative protein source for this species.

The biometric data were fully supported by the gene expression analysis of growth factors and myogenic regulatory factors. Although the insulin-like growth factors (*igfs*) are involved in several cellular process, they participate in neuroendocrine growth control with a principal role in regulation of somatic growth; their expression in muscle tissue may locally induce the expression of myogenic factors that in turn promote muscle growth [36]. Interestingly, gene expression of both *igf1* and *igf2* in muscle tissue showed an upregulation in fish fed with BSF meal, supporting the growth parameters registered during the experiment. Surprisingly, the *igf2* mRNA level was higher in juvenile zebrafish fed with V instar BSF meal, in respect to both control and to the prepupae meal. Gabillard et al. [37] demonstrated that the gene expression of *igf1* and *igf2* in fish are dependent on feeding regime; our results on *igf2* expression in juvenile zebrafish suggest that V instar and prepupae BSF meals had different effects on fish growth, probably due to their different nutritional values, as previously demonstrated [15,16].

Besides insulin-like growth factors, other key factors regulate fish growth. In particular, muscle growth is modulated by myogenic regulatory factors (MRFs) and myostatin—a negative endogenous regulator—that finely control the process until the adult. Although the effect of different diets on myogenesis has been poorly investigated, the few published studies showed that the nutrient supply could influence the expression of the genes coding for myogenic factors *myf5*, *myod,* and *myog,* thus orchestrating the process of skeletal myogenesis [38]. The observed gene expression patterns in zebrafish exposed to the two different BSF-based diets evidenced that the maintenance of fish growth is controlled by the differential regulation of precise muscle growth–related genes in juveniles and adults. While neither the V instar nor the prepupae meals were able to modulate the expression of *myod1,* at the juvenile or the adult stage, the modulation of the expression of *myf5* and *mstnb* in adults, as well as *myog* in juveniles, strongly suggests that the different nutritional properties of the two BSF meals may modulate the growth response in fish.

One of the major controversial issues in using insect-based meals in fish diet is the high level of chitin, the major constituent of the exoskeleton. It is a common idea that high chitin level inclusion in aquafeeds can negatively influence the digestibility as well as the utilization of nutrients; thus, compromising fish growth and health [4,39,40,41,42,43,44]. Previous studies reported the different chitinolytic activity of several fish species [39,45,46,47]. Our results clearly showed that the gene expression levels of chitinases were remarkably high in adult fish only. Moreover, it is worthy to note that the gene expression of all chitinases was much higher in fish fed with BSF prepupae in respect to fish fed with BSF V instar, with the latter comparable to control. These data suggest that the chitinolytic activity in zebrafish is strictly dependent on the BSF developmental stage used as feed, having prepupae meal higher chitin content compared to V instar meal [15]. In our opinion, these findings give important knowledge on the inclusion of insect in aquafeeds and open innovative scenarios for selecting the appropriate BSF-based diet for different fish species or developmental stages, thus ensuring a normal feed intake and growth in those species where the chitinolytic activity is scarce or lacking.

Several studies have investigated the effects of chitin on the immune system to elucidate its potential as an immunostimulatory additive for fish feed [48]. In general, the inclusion of chitin at low levels (from 0.01% to 2%) in diets for fish, generally activates the innate immunity [42,49,50]. Moreover, studies have investigated the effect of dietary insect meals on the immune system in fish. In yellow catfish (*Pelteobagrus fulvidraco*), a diet with 25% substitution of fishmeal with BSF larvae meal protein enhanced the immunocompetence of the fish [51]. In Atlantic salmon, the total replacement of fishmeal with BSF larvae meal had no effect on the transcription of pro-inflammatory genes in the head kidney cells; however, an effect on the transcription of antioxidant and stress related genes in the leukocytes was observed [52]. In the present study, the levels of chitin were 6.31% and 7.22% in defatted V instar larvae and defatted prepupae meals, respectively. These levels of chitin in the BSF diets might have an impact on the modulation of the expression of interleukins (*il1b* and *il6*) in juveniles, but not in adult fish; on the contrary, *tnfa* expression in adults was modulated only by BSF V instar meal, showing a peculiar response to the different BSF meals.

The effects of different BSF meals on zebrafish wellness were evaluated measuring the expression of stress-related genes (*hsp70* and *nr3c1*). Both genes were modulated by the different BSF diets exclusively in juvenile zebrafish; however, no differences were observed among the three treatments in adult fish (90 dph), showing that both BSF diets had no negative effects on adult fish. This result is in accordance with Zarantoniello et al. [13], where the effects of a full fat BSF meal inclusion of up to 50% was evaluated in 6-month adult zebrafish.

The results obtained from the zebrafish model organism can be useful for understanding the physiological and molecular responses of fish to insect-based diets that could be common to most aquaculture species. Indeed, zebrafish share the same or similar physiological response, besides developmental mechanisms and morphological features with many aquaculture species [13], thus providing useful information about the possible effects of BSF inclusion as feed ingredient in conventional aquaculture.

Taken together, our findings demonstrate that the two BSF developmental stages used as meal in zebrafish diets have different effects on fish growth and peculiar effects on immune response and modulation of chitinases. Therefore, 100% of defatted BSF V instar or prepupae meal inclusion in the zebrafish diet could overcome the limitations of using fishmeal in aquafeeds; thus, suggesting the intriguing perspective of using defatted BSF-based meals in feed formulation as valuable and sustainable alternative protein sources for relevant aquaculture fish species.

Noteworthy, the herein tested BSF meals represent the larvae and prepupae biomass obtained through the bioconversion of vegetable waste; thus, highlighting the great potential of *H. illucens* in the waste valorization process, a new agri-food biotechnology to obtain high value nutritional resources in a circular economy context.

## 5. Conclusions

Our results indicate that fishmeal can be totally replaced with defatted BSF meals in diets for zebrafish. Particularly, the defatted BSF prepupae meal can promote zebrafish growth that, in turn, is supported by myogenesis-related genes expression. In addition, this study revealed that the expression of the genes involved in enzymatic hydrolysis of chitin (*chia.2*, *chia.3*, and *chia.5*) could be modulated by BSF diets, particularly in adult zebrafish fed BSF prepupae. Finally, the expression of genes related to stress and immune response indicate that diets containing defatted BSF meals do not impair the wellness of fish. Thus, our results suggest that BSF defatted V instar or prepupae meals are promising ingredients for the replacement of fishmeal in aquafeeds.

## Figures and Tables

**Table 1:** Formulation (g Kg^−1^) and proximate composition * of experimental diets.

Ingredients	Control	V Instar	Prepupae
Fish meal	500	-	-
Defatted V instar larvae meal	-	500	-
Defatted prepupae meal	-	-	500
Soy protein concentrate	250	148.8	200
Soybean meal	140	241.2	190
Canola oil	40	40	40
Defatted rice meal	30	30	30
Vitamin/MineralPremix	30	30	30
Salt	10	10	10
Proximate composition (%)			
Crude protein	46.43	45.39	46.92
Crude lipid	9.40	9.70	9.13
Ash	22.19	9.16	9.01
NFE ^1^	13.86	24.11	18.50
Acid detergent fiber	8.12	11.64	13.60
Chitin		6.31	7.22
Dry matter	94.53	92.69	93.30
-			
Gross energy (MJ Kg^−1^)^2^	17.09	18.74	18.57
Protein/energy (g/MJ)	27.17	24.22	25.27

* Analyzed values; ^1^ Nitrogen Free Extract = 100 − (% Crude Protein + % Crude Fat + % Crude Fiber + % Moisture + % Ash); ^2^ Gross Energy (MJ Kg^−1^) = 0.0236 Crude protein + 0.0393 Crude lipid + 0.0174 Nitrogen free extract.

**Table 2:** Primer pair sequences, amplicon size, PCR efficiency (E), GenBank/Ensembl accession number, and reference related to quantitative real-time PCR analyzed genes.

Gene Name	Forward (5′-3′) Reverse (5′-3′)	Size (bp)	E (%)	GenBank	Reference
*igf-1*	GGCAAATCTCCACGATCTCTAC	198	105	ENSDART00000004717.8	[13]
	CGGTTTCTCTTGTCTCTCTCAG				
*igf-2*	CGTGTGTGGAGAAGATGGCT	156	108	ENSDART00000009642.7	This study
	ACATCTCGCTCCGACTTCAC				
*mstnb*	CGGACTGGACTGCGATGAG	174	97	ENSDART00000100386.2	[13]
	AGATGGGTGTGGGGATACTTC				
*myod1*	AGACGAGAAGACGGAACAGC	116	99	NM_131262.2	This study
	CACGATGCTGGACAGACAAT				
*myog*	CACATACTGGGGTGTCGTCC	199	94	ENSDART00000014062.7	This study
	GCCTCTGTTCCCGTTATGCT				
*myf5*	GCAGTGTTTGTCCAGCATCG	192	102	ENSDART00000112035.2	This study
	GCAAGCAGTGTGAGTAAGCGT				
*il1b*	AGGCTGGAGATGTGGACTTC	95	94	ENSDART00000185837	[13]
	GTGGATTGGGGTTTGATGTG				
*il6*	ATGACGGCATTTGAAGGGGT	115	94	ENSDART00000166112.2	This study
	TCAGGACGCTGTAGATTCGC				
*tnfα*	GGAGAGTTGCCTTTACCGCT	155	90	ENSDART00000025847.9	This study
	TGTTGATTGCCCTGGGTCTT				
*hsp70*	TCCTGACCATTGAAGACGGC	151	100	ENSDART00000124762.3	This study
	GCCCTCTTGTTCTGACTGATGT				
*nr3c1*	CTGTGTTTCGCTCCAGACCT	184	94	ENSDART00000181179.1	This study
	TCTTCAACCCATCCTTCGG				
*chia.2*	AGTGCTGCTGTATCTGCTGG	127	90	ENSDART00000164702.2	This study
	CTGTGAATCGCTCCCAAGTT				
*chia.3*	TGCCTCCAATGCCTTCAACT	122	101	ENSDART00000067817.6	This study
	TCCATCTGGCTTCCCATTACA				
*chia.5*	CACGGCTCACAGGACAACAT	160	105	NM_001110041	This study
	CATACGCAGCAAAGCCCAT				
*arp*	CATCTCGCCCTTCTCCTACG	168	101	AF134852	This study
	GCAAGAGTTGGGTAGCCGAT				
*rpl13*	ATCTCTGTTGACTCACGCCG	80	103	ENSDART00000176368.2	This study
	GTGCGGTATTCCTTCAGCCT				
*ef1-α*	CCTGCCAATGTAACCACTGA	193	95	NM_131263	This study
	TGATGACCTGAGCGTTGAAG				

**Table 3:** Survival rate and growth parameters of zebrafish (*Danio rerio*) fed with experimental diets (control, V instar, and prepupae) for 60 days.

Treatment	Control	V Instar	Prepupae
Survival rate (%)	92.50 ± 3.23 ^a^	93.75 ± 4.73 ^a^	96.25 ± 2.39 ^a^
Final total length (mm)	24.11 ± 0.64 ^b^	26.08 ± 0.98 ^ab^	28.46 ± 0.38 ^a^
Final standard length (mm)	20.14 ± 0.63 ^b^	21.84 ± 0.68 ^ab^	23.08 ± 0.26 ^a^
Height (mm)	5.38 ± 0.16 ^b^	5.71 ± 0.12 ^ab^	6.11 ± 0.09 ^a^
Final body weight (mg)	132.99 ± 10.39 ^b^	159.41 ± 13.58 ^ab^	189.70 ± 5.00 ^a^
Weight gain (mg)	127.99 ± 10.39 ^b^	154.41 ± 13.58 ^ab^	184.70 ± 5.39 ^a^
Daily growth rate (mg/day)	2.13 ± 0.17 ^b^	2.57 ± 0.23 ^ab^	3.08 ± 0.08 ^a^
Specific growth rate	5.37 ± 0.12 ^b^	5.67 ± 0.14 ^ab^	5.97 ± 0.04 ^a^
Condition factor index	1.55 ± 0.02 ^a^	1.39 ± 0.03 ^b^	1.46 ± 0.03 ^ab^

Data are expressed as mean ± S.E. Different letters indicate significant differences among treatments (*p* < 0.05; ANOVA).

## Data Availability

The data presented in this study are available within the article and/or its supplementary materials.

## Supplementary Materials

The following are available online at https://www.mdpi.com/2076-2615/11/3/720/s1. Figure S1: weight of zebrafish (*Danio rerio*) fed with experimental diets (control, V-instar, and prepupae) throughout of experiment.
