# Supplementary material for: Black Soldier Fly (Hermetia illucens) Larvae and Prepupae Defatted Meals in Diets for Zebrafish (Danio rerio)
Source: Animals (Basel). 2021 Mar 6;11(3):720. doi: 10.3390/ani11030720 (PMC7999764; doi:10.3390/ani11030720)
Supplement: Supplementary file 1 [file animals-11-00720-s001.pdf]

animals

MDPI

Article

Black Soldier Fly (*Hermetia illucens*) Larvae and Prepupae Defatted Meals in Diets for Zebrafish (*Danio rerio*)

Carlos F. C. Lanes, Fabio A. Pedron, Giovani T. Bergamin, Andressa L. Bitencourt, Brenda E. R. Dorneles, Jessica C. V. Villanova, Kimberly C. Dias, Kristian Riolo, Sabrina Oliva, Domenico Savastano and Alessia Giannetto

Supplementary Materials

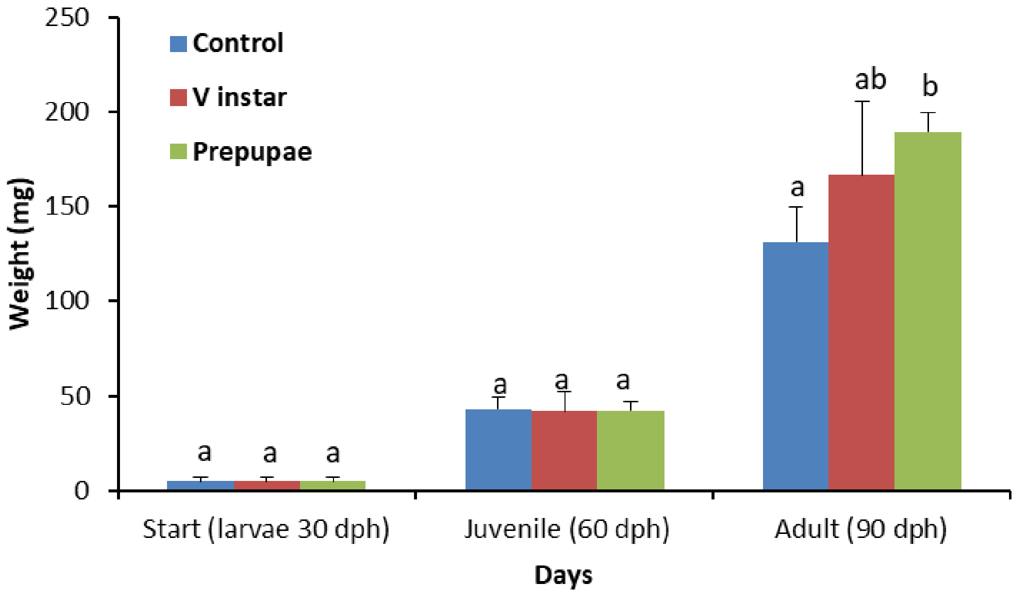

Figure S1. Weight of zebrafish (*Danio rerio*) fed with experimental diets (control, V-instar, and prepupae) throughout of experiment.
